# Visuo-Spatial Imagery Impairment in Posterior Cortical Atrophy: A Cognitive and SPECT Study

**DOI:** 10.3233/BEN-2011-0279

**Published:** 2011-05-23

**Authors:** Simona Gardini, Letizia Concari, Salvatrice Pagliara, Caterina Ghetti, Annalena Venneri, Paolo Caffarra

**Affiliations:** ^1^Department of NeuroscienceUniversity of ParmaParmaItaly; ^2^Outpatient Clinic for the Diagnosis and Therapy of Cognitive DisordersAUSLParmaItaly; ^3^Struttura di Fisica SanitariaAzienda Ospedaliera UniversitariaParmaItaly; ^4^Clinical Neuroscience CentreUniversity of HullUnited Kingdom and S. Camillo Hospital (I.R.C.C.S)VeniceItaly

**Keywords:** Posterior cortical atrophy, imagery, spatial abilities, visual process, dementia, rCBF

## Abstract

This study investigated the cognitive profile and the cerebral perfusion pattern in a highly educated 70 year old gentleman with posterior cortical atrophy (PCA). Visuo-perceptual abilities, spatial memory, spatial representation and navigation, visuo-spatial mental imagery, semantic and episodic-autobiographical memory were assessed. Regional cerebral blood flow (rCBF) was imaged with SPECT. Cognitive testing showed visual-perceptual impairment, apperceptive visual and landmark agnosia, topographical disorientation with way-finding deficits, impaired map learning and poor mental image generation. Semantic memory was normal, while episodic-autobiographical memory was impaired. Reduced rCBF was found mainly in the right hemisphere, in the precentral gyrus, posterior cingulate and middle temporal gyri, cuneus and precuneus, in the left superior temporal and lingual gyri and in the parahippocampus bilaterally. Hypoperfusion in occipito-parietal regions was associated with visuo-spatial deficits, whereas deficits in visuo-spatial mental imagery might reflect dysfunction related to hypoperfusion in the parahippocampus and precuneus, structures which are responsible for spatial and imagery processing. Dissociating performance between preserved semantic memory and poor episodic-autobiographical recall is consistent with a pattern of normal perfusion in frontal and anterior temporal regions but abnormal rCBF in the parahippocampi. The present findings indicate that PCA involves visuo-spatial imagery deficits and provide further validation to current neuro-cognitive models of spatial representation and topographical disorientation.

## 1. Introduction

Posterior cortical atrophy (PCA) is a rare form of progressive degenerative dementia which at onset is characterized by very invalidating visual impairments. The first report of PCA described five patients presenting with an unusual form of Alzheimer's disease (AD), in which the primary symptoms at onset were visual agnosia, alexia, Balint's syndrome (ocular fixation, optic ataxia, simultagnosia), Gerstmann's syndrome (agraphia, acalculia, digital agnosia and left/right disorientation) and transcortical sensory aphasia [3]. Levine et al. [23] reported the case of a patient with a similar cognitive profile which worsened with the progression of the disease, whose initial symptoms were visual impairments with sparing of memory.

The nosological definition of PCA remains far from being completely understood, and it is not clear whether this clinical syndrome is a form of AD with an atyp-

ISSN 0953-4180/11/$27.50 © 2011 – IOS Press and the authors. All rights reserved ical clinical presentation or whether this form of neurodegeneration represents a distinct nosological entity [33]. Tang-Wai et al. (2004) described the clinical, genetic and neuropathological features of PCA in forty patients [38]. They reported that the cognitive deficits are characterised by simultagnosia, visual fields defects, acalculia, alexia, anomia and preserved insight early in the course of the disorder. The mean age of onset of the disease was 60.5 years (SD 8.9) with prevalence of females. Genetic analysis showed similar findings to typical AD, with a similar proportion of APOE $\varepsilon$4 and tau haplotypes. In most cases the neuropathological features were similar to those of typical AD with a higher density of neurofibrillary tangles in visual structures, such as Brodmann areas 17 and 18, and lower density in the hippocampus. Two patients who developed asymmetric parkinsonism and ideomotor apraxia had histopathological findings compatible with corticobasal degeneration. Features typical of other pathologies were also found, such as Lewy body dementia or Creutzfeldt-Jacob disease which phenotypically presented as PCA. The authors concluded that PCA might be considered a distinct form of dementia separated from AD.

Diagnostic criteria for PCA were proposed by Mendez et al. [30] and then later redefined by Tang-Wai et al. [38]. These authors proposed that the core features of PCA should be an insidious onset and gradual progression, the presence of disabling visual deficits in the absence of any ocular disease, preserved anterograde memory, insight and awareness of illness, absence of tumour, stroke, parkinsonism or visual hallucinations. Other features that might be found are simultagnosia, visuoconstructive apraxia, visual field deficits, topographical disorientation, and some elements of the Gerstmann's syndrome. Further features might be presenile onset, alexia, ideomotor or dressing apraxia, and prosopoagnosia. These clinical characteristics should be associated with a pattern of structural and/or functional brain dysfunction in parietal and/or occipital regions.

PCA presents the neuropathological features typical of AD [2], but these are located in different cerebral areas [43]. In typical AD neurofibrillary tangles and neuritic plaques are usually located primarily in memory-related areas, such as hippocampal and medial-temporal cortex. In PCA these neuropathological features are mainly situated in parieto-occipital areas which are responsible for visual processing [39].

Neuroanatomical studies showed that PCA is characterized by a higher degree of atrophy in parieto-occipital regions [38]. Functional imaging has shown that when compared to healthy controls PCA patients had lower levels of metabolism mainly in frontal (the frontal eye field area) and parieto-occipital regions, whereas a defined region of hypometabolism in the right parieto-occipital areas was clearly evident when compared to typical AD [32]. Schmidtke et al. (2005) described the brain metabolism pattern of six PCA patients [36]. At the individual level there was a variable involvement of temporal, occipital and frontal areas, while all patients showed a deficit in the parietal associative cortex. Comparing the MRI data of PCA and typical AD, Whitwell et al. (2007) found that PCA was characterised by a higher degree of atrophy in visual associative regions, while in typical AD greater atrophy was found in the hippocampal cortex [41]. These anatomical differences suggested that the clinical differences in PCA and typical AD stem from the impairment of distinct brain regions.

In PCA two different phenotypes have been described reflecting either damage of the dorsal or ventral visual pathways [17,34,37]. Degeneration of structures in the dorsal occipito-parietal stream, the "where pathway", is associated with visuo-spatial deficits, Balint's syndrome, transcortical sensory aphasia, apraxia, elements of the Gerstmann's syndrome, whereas the degeneration of areas in the ventral occipito-temporal stream, the "what pathway", causes alteration in the visual perception of objects, apperceptive visual agnosia, alexia and prosopoagnosia.

The ventral and dorsal pathways contain both primary and associative visual areas [31] Associative visual areas were found to be involved in the retrieval of information from remote memory [25] as well as in mental image generation [9,16,21]. It is plausible to assume, therefore, that neurodegeneration in PCA might also disrupt aspects of remote autobiographical memory and of mental imagery, cognitive abilities which have not been investigated by previous studies.

The present study aimed to investigate the cognitive and neuroimaging correlates of PCA, focusing on those cognitive abilities which have been neglected in previous assessments of this syndrome, such as spatial representation, visual imagery and episodic autobiographical memory.

This study also aimed to add further evidence to current models of spatial cognition and topographical disorientation which have associated cognitive deficits with specific cerebral lesions.

## 2. Methods

### *2.1. Case report*

A 70 year old gentleman, retired University professor with 18 year of education, came to our attention for the first time in December 2008 because of progressive visuo-spatial deficits. He reported progressive difficulties in motor coordination during simple every day life activities such as getting dressed, visual memory deficits, disgraphia, all dating as far back as three years. When reading books or newspapers he was able to follow individual lines although with some difficulty. He had also been experiencing spatial disorientation for about one year. For approximately two years since symptoms onset, he remained able to go to work by bus independently. Neurological examination was unremarkable, except for a modest degree of hesitancy in the sitting down manoeuvre or when walking in a narrow space.

### *2.2. Neuropsychological assessment*

The standard neuropsychological battery included the following tests: Mini-Mental State Examination (MMSE), Activity of Daily Living (ADL), Instrumental Activities of Daily Living (IADL), Raven Coloured Progressive Matrices 47 (PM47), verbal (letter and category) fluency tasks, Boston naming test, Benton face recognition test, Albert test, digit span, immediate visual memory and Rey Auditory Verbal Learning Test (RAVLT, immediate and delayed), Birmingham Object Recognition Battery (BORB, subtest 7, a minimal features view task), copying of line drawings to evaluate constructional apraxia, ideomotor apraxia test and Stroop test.

Part of the Visual Object and Space Perception battery (incomplete letters, dot counting, position discrimination, number location, shape detection screening test and cube analysis) (VOSP, Warrington and James, 1991 [40]) was used to assess visuo-perceptual abilities.

Testing of temporal orientation and spatial abilities included simple questions about temporal coordinates and familiar environments, e.g. his house, testing of navigation abilities, the route he was used to take to go to work, drawing a map of this route. Moreover, a map learning test was administered: the patient was asked to learn a simple map and then to locate the verbal labels of a set of landmarks in the correct position [12]. The ability to generate mental images was tested asking the patient to mentally evoke an image of some concrete objects and to verbally describe them [11]. Concrete object recognition was investigated using a computerised version of the Snodgrass and Vanderwart test [35]. The presence of visual and digital agnosia and of simultagnosia was ascertained. Language functions (e.g. confrontation naming, writing, reading) were evaluated using the neuropsychological examination for aphasia battery (ENPA, [10]). Calculation skills were also tested using the calculation component of the ENPA battery. Reaching for objects, perception of movement, stereoscopy and left and right orientation were also evaluated as part of this battery. Visual memory for familiar faces was assessed by asking the patient to identify photos of familiar individuals in a set including also unknown people. Semantic memory was measured administering part of the Laiacona et al. (1993) [22] test while the test of Ivanoiu et al. (2006) [18] was used to test semantic and episodic autobiographical memory. The presence of mood disorders, anosognosia, strategies of compensation to cope with the cognitive deficits and related social disability was also ascertained.

### *2.3. Neuroimaging acquisition and analysis*

The patient underwent cerebral MRI and SPECT imaging. For SPECT, the scan of the patient was compared with those of a group of age matched healthy controls. The patient and the control group were injected with 740 MBq of $^{99m}$Tc-HMPAO. One hour after the injection they underwent a SPECT scan with a dual head gamma camera (Adac Genesys Vertex) equipped with FBLF (Fan Beam Long Focus) collimators, with a rotation radius of 15–17 cm. The acquisition matrix was $128 \times 128 \times 16$ with a pixel size of 4.6 mm, the number of projections were 64, with an acquisition time per projection of 40 seconds. The raw data were reconstructed using a Filter Back Projection technique with a Butterworth 5 order filter and a cut-off frequency of 0.4 cycles cm$^{-1}$. After reconstruction the images were corrected for attenuation with a Chang first order technique and a linear attenuation coefficient of 0.11 cm$^{-1}$. To remove any background signal the brains were masked from the images using a 3D ellipsoid-shaped region of interest (ROI). To eliminate low intensity background noise and brain edge image artifacts caused by any partial volume effects, images were cut-off twice below the threshold of 30% of the maximum voxel values and then 70% of the mean voxel value. These images were saved in DICOM format and then individually normalised to the cerebellum counts using the ImageJ 1.29x software package (National Institutes of Health, USA). This procedure was followed to ensure that the counts in the cerebellum of each individual were the same prior to any processing, which should in turn result in increased sensitivity and specificity of the analysis. The software ImageJ was also used to convert the images into ANALYZE format for subsequent analysis.

The patient's regional cerebral blood flow (rCBF) image was compared with those of six healthy age matched controls (mean age = 64.16, SD = 8.30; mean education = 10.33, SD = 6.02; three males and three females) using SPM2, (Wellcome Department of Imaging Neuroscience, UCL, London, UK). Differences in rCBF between the patient and the control sample were assessed on a voxel-by-voxel whole-brain basis using independent sample t-test with education included as confounding variables. Statistical significance level was set at $p < 0.001$ corrected.

## 3. Results

### 3.1. Cognitive testing

The patient showed visuospatial neglect for the left space. Standard neuropsychological assessment showed a cognitive profile compatible with a diagnosis of PCA, with visuo-spatial impairment, spatial disorientation, difficulty in recognition of visual objects and faces, dysgraphia, calculation deficits and visuo-constructive and ideomotor apraxia (see Table 1).

The patient performed poorly on several of the VOSP subtests, i.e. dot counting, incomplete letters, number location and cube analysis. He had a relatively better performance on two subtests, i.e. position discrimination and shape detection screening (see Table 1).

He was disoriented in time, being unable to remember the exact date, and failed in the description of his house and familiar places, showing landmarks agnosia. He could provide a relatively good description of the room where he worked, however. When asked, he was unable to describe the relative position of the rooms in his apartment, showing disorientation even for familiar space.

By the time of his referral to our service, he had already lost his spatial navigation capacity, could not go to work independently and became even unable to give a verbal description of this well learned route or to draw this habitual path on the city map. The description of

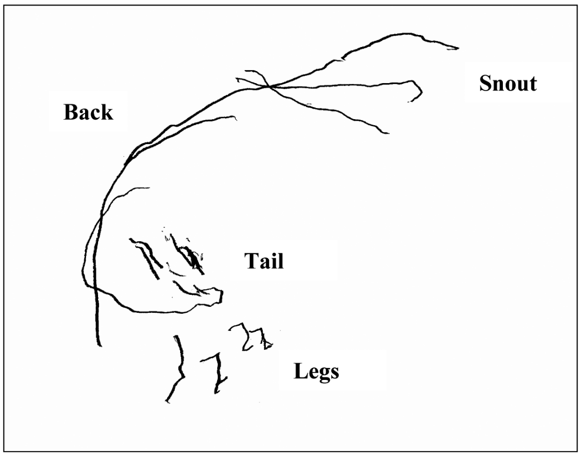

Fig. 1. Spontaneous drawing of a dog (example of exploded drawing).

his office he could offer was extremely poor. Learning of a map was also impaired as he failed in placing the verbal labels referring to specific landmarks in the correct position.

The patient showed visual apperceptive agnosia in the recognition of complex objects [23]: while he could recognise simple objects, he had difficulties in the identification of multipart objects which required the assembling of several visual details in order to generate their global shape. His performance in mental image generation also showed a similar complexity effect. He showed simultagnosia and tactile agnosia for complex objects, but occasionally also for simple objects, and he had digital agnosia.

His performance on the E.N.P.A. test for aphasia indicated the presence of deficits in confrontation naming, comprehension, writing and drawing. He drew only individual details of objects scattered on the paper, and was unable to combine them to form a complete image (see Fig. 1). This type of drawing disorder has been defined as "exploded drawing" by McMonagle and Kertesz, (2006) [28].

He was severely impaired in writing. He wrote in a distorted way, was unable to write on a straight horizontal line (see Fig. 2) and sometimes produced unrecognizable words.

Reading and spelling were spared. He showed no optic ataxia as the reaching for objects in space was preserved. Perception of moving objects was also maintained. He had normal stereoscopic vision, normal perception of colours and was able to recognise left/right orientations.

The patient was unable to recognise familiar faces, both belonging to his family and people who had be-

Table 1
The patient's scores on the tests included in his standard and experimental assessments (abnormal scores are reported in bold)

| Neuropsychological test | Raw score | Corrected score | Equivalent score | Cut off |
|---|---|---|---|---|
| *Mini mental state examination (MMSE)* | **20/30** | **18/30** | | |
| Activity daily living (ADL) | 5/6 | 5/6 | | |
| Instrumental activities of daily living (IADL) | 3/5 | 3/5 | | |
| Mental deterioration battery (MDB) | | | | |
| *Progressive Matrices (PM 47)* | **10** | **8.05** | **0** | |
| Verbal fluency | 35 | 28.15 | 3 | |
| *Immediate visual memory* | **11** | **10.05** | **0** | |
| Auditory verbal learning test (RAVLT) | | | | |
| Immediate | 35 | 35.4 | 2 | |
| Delayed | 5 | 5.45 | 1 | |
| *Constructional apraxia* | **3** | **2.35** | **0** | |
| Laiacona's semantic memory test | 465 | | Total Score<br>M = 469.12; DS = 8.05 | |
| Semantic fluency | 32 | 30.5 | 2 | |
| *Boston naming test* | **12** | | | **16** |
| *Albert's test* | | | | |
| *Omission right* | **10** | | | |
| *Omission left* | **28** | | | |
| Digit span | 5 | 4.5 | 2 | |
| BORB – subtest 7 | 17 | | M = 23.3, DS = 2 | |
| *Ideomotor apraxia* | **12** | **12** | **0** | |
| Stroop test | | | | |
| Interference time | 38.5 | 33.62 | 1 | |
| Interference errors | 4 | 3.62 | 1 | |
| *Face recognition – Benton test short form* | **< 25** | **< 27** | | |
| *Test for aphasia (E.N.P.A.)* | | | | |
| Repetition words | 10 | 10 | | 8.8 |
| non words | 5 | 5 | | 2.0 |
| sentence | 3 | 3 | | 3 |
| Reading words | 10 | 10 | | 6.4 |
| non words | 5 | 5 | | 4.0 |
| sentence | 2 | 2 | | 1.3 |
| *Writing* words | **3** | **2.7** | | **6.3** |
| *non words* | **1** | **0.6** | | **1.4** |
| *sentence* | **0** | **0** | | **0.6** |
| *Denomination writing nouns* | **0** | **0** | | **2.7** |
| *oral verb* | **3** | **2.6** | | **6.1** |
| *written verb* | **0** | **0** | | **3.0** |
| *Comprehension visual words* | **16** | **16** | | **17** |
| *auditory sentences* | **6** | **6** | | **11.6** |
| *visual sentences* | **N/A** | **5.1** | | **11.3** |
| *Number reading* | **5** | **5.7** | | **7.6** |
| *dictate* | **6** | | | **6.3** |
| *Calculation addition* | **N/A** | | | **2.2** |
| *subtraction* | **N/A** | | | **1.0** |
| *multiplication* | **N/A** | | | **1.4** |
| *Visual object and space perception (VOSP)* | | | | |
| *Incomplete letters* | **N/A** | | | |
| *Dot counting* | **N/A** | | | |
| Position discrimination | 6/8 | | | |
| *Number location* | **N/A** | | | |
| Shape detection | 8/18 | | | |
| *Cube analysis* | **N/A** | | | |

Table 1, continued

| Neuropsychological test | Raw score | Corrected score | Equivalent score | Cut off |
|---|---|---|---|---|
| *Map learning* | | | M = 4.25; SD = 1.2 | |
| *Number of landmark* | **0/8** | | | |
| *Test of autobiographical memory* | | | | |
| *Semantic score* | | | | |
| *childhood* | **12/24** | | M = 21.4; SD = 1.8 | |
| *early adulthood* | **13/24** | | M = 20.3; SD = 1.9 | |
| *late adulthood* | **6/24** | | M = 17.4; SD = 3.3 | |
| *last 5 years* | **9/24** | | M = 20.9; SD = 1.7 | |
| *Episodic cued* | | | | |
| *childhood* | **12/30** | | M = 24.0; SD = 4.3 | |
| *early adulthood* | **15/30** | | M = 24.7; SD = 2.5 | |
| *late adulthood* | **10/30** | | M = 22.5; SD = 3.9 | |
| *last 5 years* | **12/30** | | M = 23.3; SD = 2.4 | |
| *Name fluency* | | | | |
| *childhood* | **3** | | M = 21.3; SD = 10.1 | |
| *early adulthood* | **7** | | M = 17.2; SD = 7.1 | |
| *late adulthood* | **1** | | M = 15.7; SD = 8.5 | |
| *last 5 years* | **1** | | M = 16.0; SD = 9.8 | |
| *Episodic free* | | | | |
| *childhood* | **1.5** | | M = 29.8; SD = 11.1 | |
| *early adulthood* | **3** | | M = 26.1; SD = 9.4 | |
| *late adulthood* | **0** | | M = 19.4; SD = 9.1 | |
| *last 5 years* | **0** | | M = 21.9; SD = 10.7 | |

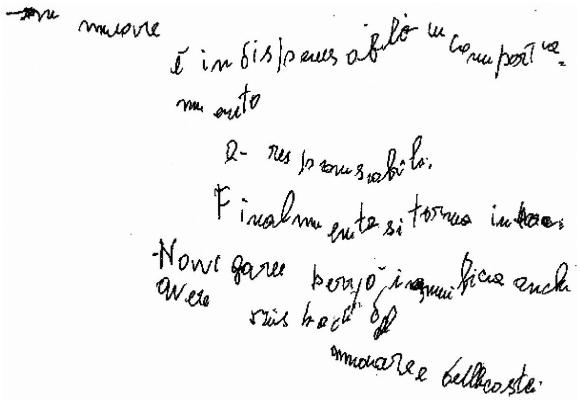

Fig. 2. Visuo-constructive agraphia in writing to dictation.

come involved in his rehabilitation programme. Semantic memory, investigated with the Laiacona test was in the normal range. Autobiographical memory was poor, and episodic recollection of past personal events was greatly impaired. He retained semantic personal memories and was able to recall semantic autobiographical information, but without any specific details about events, or any detailed spatial and temporal context.

The patient showed complete awareness of the disease from the onset of initial symptoms and his mood became negatively affected due to his severe limitations in daily activities related to the visual and spatial impairment.

During testing, he tried to compensate for his visuo-perceptual deficits, using verbal and semantic strategies. For example, when asked to say the colour of the skin of his wife, he was unable to answer. He tried to compensate using some general semantic knowledge to provide an answer and he said that as his wife was born in a city on the west coast of Italy, and since there women usually have quite dark skin, he then came to the inaccurate conclusion that she should also have quite dark skin. This example suggests that he was unable to recall visual characteristics of familiar people, even if he was not impaired in colour recognition. In general, he tended to be prolix and responded simple questions with long answers not always related to the original issue.

He was not keen to engage in social activities, tended to stay alone and experienced high levels of fatigue and anxiety when faced with new activities.

### 3.2. Neuroimaging results

MRI showed more pronounced atrophy in the right parieto-occipital lobe, in the absence of markers of cerebrovascular lesions (see Fig. 3).

The analysis of SPECT scans showed significant hypoperfusion primarily in the right hemisphere, in the precentral (BA 4), cingulate (BA 23 and 31) and middle temporal (BA 37) gyri, in the cuneus (BA 7) and precuneus (BA 7). Hypoperfusion was also seen in

Table 2
Areas of significant decrease of rCBF in the PCA patient compared with controls

| Brain area | Left/Right | Brodmann area (BA | Z value at local maximum | Talairach coordinates x | y | z |
|---|---|---|---|---|---|---|
| Precentral gyrus | R | 4 | 4.95 | 30 | −27 | 51 |
| Cingulate gyrus | R | 23 | 4.72 | 10 | −26 | 33 |
| | R | 31 | 4.51 | 22 | −45 | 35 |
| Parahippocampal gyrus | L | 30 | 4.58 | −26 | −54 | 5 |
| | R | 19 | 4.00 | 38 | −45 | −3 |
| | R | 19 | 3.89 | 22 | −56 | −4 |
| Lingual gyrus | L | 19 | 4.37 | −24 | −66 | 3 |
| Superior temporal gyrus | L | 39 | 4.09 | −28 | −53 | 23 |
| Cuneus | R | 7 | 4.22 | 8 | −74 | 33 |
| Precuneus | R | 7 | 3.38 | 8 | −63 | 31 |
| Middle temporal gyrus | R | 37 | 3.79 | 44 | −66 | 7 |

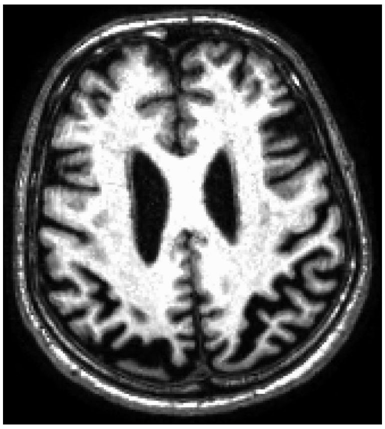

Fig. 3. Magnetic resonance imaging of the patient.

the left hemisphere in the superior temporal (BA 39) and lingual (BA 19) gyri and bilaterally in the parahippocampus (see Table 2, Figs 4 and 5).

## 4. Conclusion

The present study examined the cognitive and neurophysiological profiles of a patient presenting with cognitive decline associated with posterior cortical atrophy. He presented with an insidious onset and slowly progressive decline of visual and cognitive abilities. He experienced progressive visuo-perceptual deficits, apperceptive visual agnosia and tactile, digital and landmark agnosia, visuo-spatial disgraphia, acalculia, visuo-constructional and dressing apraxia as well as poor spatial memory and topographical disorientation with deficits in way-finding. Visual object recognition was impaired especially with complex and multipart stimuli suggesting a degree of simultagnosia. Visual imagery and mental representation processes were impaired and resulted in impairment in his ability to generate mental images of objects and visuo-spatial scenes. Colour perception was relatively preserved. There was a clear dissociation between episodic and semantic autobiographical recollection with relative preservation of the semantic aspects of his personal memories but severe loss of the episodic aspects of his personal recollections causing loss of the specific spatial and temporal details of events. SPECT data showed hypoperfusion mainly in the right hemisphere, in occipital areas (e.g. the lingual gyrus), medial-temporal (parahippocampus) and parietal regions (cuneus and precuneus).

The spectrum of deficits observed in this patient was similar to those reported in the literature. Mental imagery skills were also tested and we were able to demonstrate that in this syndrome deficits might extend to mental representations of the visual and spatial world. To our knowledge, a finding of this kind has never been reported before in similar cases of progressive posterior cortical atrophy. Symptoms reflected dysfunction of occipital, parietal and temporal areas and full involvement by the neurodegenerative process of both dorsal and visual streams [31]. The finding of additional hypoperfusion in the parahippocampal regions suggests progression of neurodegeneration to regions involved in memory processes and justifies the observed decline of spatial memory and episodic memory, further exacerbated by hypoperfusion in the precuneus which might also be responsible for the visual imagery deficits experienced by the patient [15]. The precuneus is a structure known to provide support in the generation of mental images related to episodic events, and it is considered a key structure which plays a role across imagery and memory [13,14]. Tactile agnosia was also present and might be related to damage in the posterior parietal cortex, typically involved in the processing of tactile information [5]. Colour, depth and motion perception was normal.

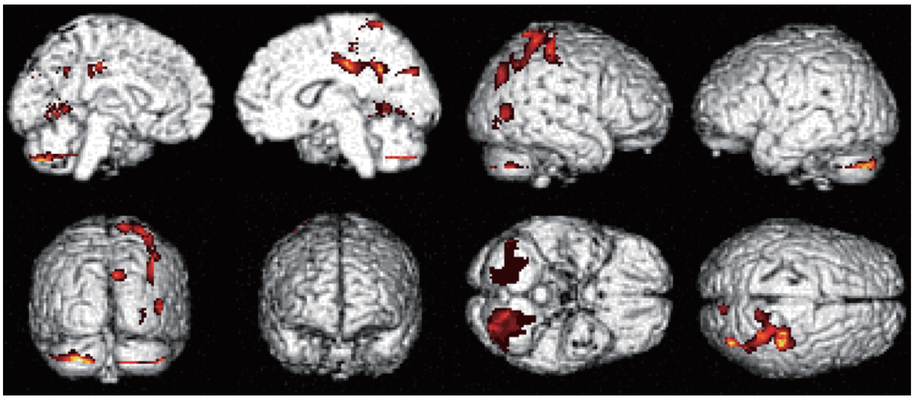

Fig. 4. Areas of significantly lower rCBF values in the patient than controls mainly located in right parieto-occipital cortex.

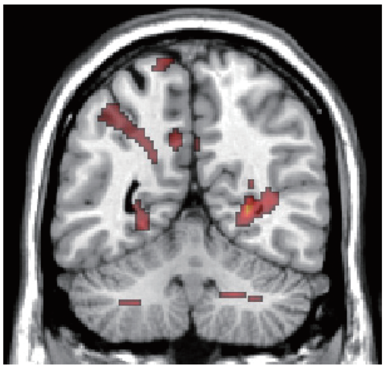

Fig. 5. Significant hypoperfusion seen in the parahippocampal cortex bilaterally in the patient.

The patient showed severe episodic autobiographical memory deficits which might be explained by parahippocampal and precuneus damage. The parahippocampal gyrus is involved in the retrieval of episodic autobiographical memories [25,42], of episodic memory for visual scenes [19,20] and does also play a role in the mental generation of episodic autobiographical images [14]. The observed dissociation between spared semantic memory and impaired episodic-autobiographical memory might be associated with a pattern of normal perfusion in frontal and anterior temporal regions [8], but abnormal rCBF in the parahippocampus bilaterally, which supports the retrieval of spatio-temporally coded life events [25,42].

Severe impairment of spatial processing was also experienced by the patient. Both hypoperfusion in the parahippocampus and in the precunes were the underlying source of this impairment, as there is evidence that the parahippocampus is associated with processing of spatial context [4], with exploration and integration of salient objects in the environment [27], with mental navigation in a setting rich of routes and landmarks [29] and with supporting topographical memory [24]. The parahippocampus receives connections from posterior parietal areas and from the dorsal visual processing pathway [4]. These connections are crucial to support the processing of spatial information in visual scenes and the resulting activity can be controlled by attention [6]. The retrieval of the spatial context of events would, therefore, require the involvement of both parahippocampal gyrus and precuneus [7]. Indeed, the precuneus would be instrumental in constructing an internal representation of large-scale environments [27], and it has been found activated during topographical memory encoding [26].

Theoretically these findings can be explained within a framework for spatial processing that suggests that medial-temporal regions would be responsible for the storage of allocentric representations, while the posterior parietal cortex would be involved in the formation of egocentric representations, maintained in the temporo-parietal cortex and the precuneus contributing to the generation of a mental visual representation of space [7]. Progressive damage of parahippocampal and parietal areas might preclude the formation of a mental map of space and hamper actual effective navigation.

The pattern of cognitive and neurophysiological deficits observed in the patient substantiates the taxonomy of topographical disorientation deficits proposed by Aguirre and D'Esposito in 1999 [1]. The authors reviewed the published studies on spatial navigation deficits and derived four types of topographical disorientation characterised by specific brain lesion sites and patterns of cognitive impairment. In particular, they suggested that lesions in posterior parietal regions would be associated with egocentric disorientation which is characterised by the inability to represent the position of external objects in reference to self; lesions in posterior cingulate would be related with heading disorientation such as the impairment in representing the direction of a given orientation with respect to the external environment. Two other disorder labels were suggested, i.e. landmark agnosia (difficulty in representing the appearance of salient landmarks) and anterograde disorientation (the inability to create new representations based on environmental information), associated respectively with lesions in the lingual gyrus and in the parahippocampus. Our patient had dysfunction in all the above mentioned areas and he experienced the described topographical deficits.

In conclusion, the present study has achieved two main objectives. Firstly, it has highlighted the presence of impairments in aspects of cognition which had not been explored before in patients with PCA, such as visual imagery and episodic autobiographical memory. Secondly, it has provided evidence in support of current models of spatial cognition [7] and taxonomy of topographical disorientation [1].

## Acknowledgements

The authors thank the patient and his family for their cooperation in the study. This study was supported by a grant from the Fondazione Cassa di Risparmio of Parma and Piacenza to Paolo Caffarra.
